# Accuracy of EEG Biomarkers in the Detection of Clinical Outcome in Disorders of Consciousness after Severe Acquired Brain Injury: Preliminary Results of a Pilot Study Using a Machine Learning Approach

**DOI:** 10.3390/biomedicines10081897

**Published:** 2022-08-05

**Authors:** Francesco Di Gregorio, Fabio La Porta, Valeria Petrone, Simone Battaglia, Silvia Orlandi, Giuseppe Ippolito, Vincenzo Romei, Roberto Piperno, Giada Lullini

**Affiliations:** 1 UO Medicina Riabilitativa e Neuroriabilitazione, Azienda Unità Sanitaria Locale, 40133 Bologna, Italy; 2 IRCCS Istituto delle Scienze Neurologiche di Bologna; 3 Centro Studi e Ricerche in Neuroscienze Cognitive, Dipartimento di Psicologia, Alma Mater Studiorum—Università di Bologna, Campus di Cesena, 47521 Cesena, Italy; 4 Dipartimento di Psicologia, Università di Torino, 10124 Torino, Italy; 5 Department of Electrical, Electronic and Information Engineering “Guglielmo Marconi”, University of Bologna, Viale Risorgimento, 2, 40136 Bologna, Italy

**Keywords:** disorders of consciousness, traumatic brain injury, electroencephalography, brain plasticity and connectivity, post-anoxic coma, severe acquired brain injury, acquired brain damage, linear discriminant analyses, brain functional impairment, neurocognitive disorders

## Abstract

Accurate outcome detection in neuro-rehabilitative settings is crucial for appropriate long-term rehabilitative decisions in patients with disorders of consciousness (DoC). EEG measures derived from high-density EEG can provide helpful information regarding diagnosis and recovery in DoC patients. However, the accuracy rate of EEG biomarkers to predict the clinical outcome in DoC patients is largely unknown. This study investigated the accuracy of psychophysiological biomarkers based on clinical EEG in predicting clinical outcomes in DoC patients. To this aim, we extracted a set of EEG biomarkers in 33 DoC patients with traumatic and nontraumatic etiologies and estimated their accuracy to discriminate patients’ etiologies and predict clinical outcomes 6 months after the injury. Machine learning reached an accuracy of 83.3% (sensitivity = 92.3%, specificity = 60%) with EEG-based functional connectivity predicting clinical outcome in nontraumatic patients. Furthermore, the combination of functional connectivity and dominant frequency in EEG activity best predicted clinical outcomes in traumatic patients with an accuracy of 80% (sensitivity = 85.7%, specificity = 71.4%). These results highlight the importance of functional connectivity in predicting recovery in DoC patients. Moreover, this study shows the high translational value of EEG biomarkers both in terms of feasibility and accuracy for the assessment of DoC.

## 1. Introduction

After acute brain injury and coma, a large number of surviving patients develop severe disorders of consciousness (DoC), such as unresponsive wakefulness syndrome (UWS) or minimally conscious state (MCS). UWS patients preserve basic functions, such as eye-opening and reflexive movements [1,2,3], but remain unresponsive to the external environment. MCS patients instead show minimal but reliable behavioral evidence (i.e., visual fixation or visual pursuit, verbalizations, etc.) of self and environmental consciousness [4,5]. UWS and MCS can be persistent states or can evolve toward varying degrees of recovery of consciousness. The assessment of the rehabilitative potential and the prediction of the possible clinical outcome of DoC patients are crucial for clinical and ethical reasons, and they allow for the identification of rehabilitative needs and the design of customized rehabilitative programs. Although standard clinical scales and innovative neurophysiological methods can help diagnose and predict the clinical outcome in DoC patients, these still represent complex challenges for clinicians. Indeed, even the distinction among UWS, MCS, and the emergence from MCS is based on clinical and behavioral evidence which might be hard to identify.

Behavioral and clinical scales such as the Coma Recovery Scale (CRS-R) and the Glasgow Outcome Scale (GOS) provide criteria for the diagnosis of DoC [6,7] and allow for longitudinal monitoring of the behavioral responsiveness of these patients. However, several fMRI and EEG studies [8,9,10,11,12] showed that ~15–20% of DoC patients with no evidence of overt behavioral responsiveness may nevertheless show signs in the brain activity of covert consciousness. Moreover, the prediction of patients’ clinical outcomes, solely based on clinical scales, is not reliable (misdiagnosis rate up to 40% [13,14,15]), especially in DoC after traumatic etiologies, where the prediction of the clinical outcome can be even less accurate compared to post anoxic/ischemic etiologies [13]. Therefore, accurate diagnostic tools that rely on brain activity [12] and on patients’ characteristics have become a critical need. In this sense, the EEG can be a useful tool. Indeed, the EEG already has numerous applications in clinical settings for the prediction of recovery in neurological patients [16] and after the application of specific protocols [17,18]. Moreover, new robust statistical methodologies, such as machine learning, have been already implemented in EEG studies to help with clinical and rehabilitative decision making [16,17,19,20,21]. Accordingly, recent studies investigated the sensitivity and accuracy of quantitative EEG (qEEG) and EEG-based functional connectivity measures to predict the clinical outcome in DoC patients [20,21,22]. Specifically, patients with reactive EEG signal to external stimuli, larger EEG amplitudes, and stronger activity in the higher-frequency bands (i.e., alpha 7–13 Hz and beta 14–25 Hz) are more likely to have a positive outcome after 3–6 months [23,24,25].

Furthermore, the complexity of the EEG signal in terms of diversity and integration is considered an important proxy of the consciousness level [26,27]. Indeed, measures that quantify the complexity of information content in the brain activity, such as the evoked EEG activity after transcranial magnetic stimulation [28,29,30] and the permutation entropy (PeEN), is reduced in patients with DoC [31,32,33,34] and in patients with worse clinical outcome [21,35]. Another important finding suggests that the level of functional connectivity in the brain, reflecting information sharing within different cortical areas [36], is an accurate index for discriminating different degrees of consciousness and predicting recovery in DoC patients [20,21,22]. In particular, EEG-based measures of connectivity are larger in healthy participants compared to DoC patients and in MCS compared to UWS patients [20,21,37,38]. Lastly, DoC patients with stronger global connectivity [21], anterior forebrain connectivity [39,40], and thalamic–cortical connectivity [41] can have a better long-term outcome in terms of disability and level of consciousness [42].

However, most of the studies reported above recorded brain activity using different paradigms and EEG configurations or high spatial sampling of scalp electrodes (i.e., high-density EEG). Albeit promising, these methodologies are often time-demanding, requiring specific protocols and apparatuses, thus preventing a large-scale implementation in clinical settings where, more likely, only standard EEGs with the 10–20 EEG montage density are available. Thus, implementing new methodologies with high feasibility in clinical settings becomes fundamental in terms of clinical usefulness and to exploit the potential of EEG-based measures in predicting the clinical outcome of DoC patients. From this perspective, the recent literature has started to highlight the relevance of quantitative EEG measures and specifically how the dominant frequency [23] and alpha power [22] extracted from standard clinical EEG can predict clinical outcome in DoC patients. On this basis, in the present study, we hypothesized that quantitative measures and measures of functional connectivity derived from standard clinical EEG can reliably predict the clinical outcome of DoC patients after traumatic and nontraumatic brain injury.

To test this hypothesis, we set two steps of analyses with the main goal of identifying EEG predictors of functional outcome in DoC patients. The first step of the study was to identify and characterize the role of EEG biomarkers in discriminating the etiology and the clinical outcome in DoC patients. Specifically, we identified those EEG measures which can discriminate DoC patients as a function of the etiology of their brain injury (i.e., traumatic or nontraumatic) and clinical outcome 6 months after the injury (i.e., improved patients or nonimproved patients) using a standard clinical resting-state EEG. The study’s second step was to investigate the accuracy of the EEG biomarkers to predict clinical outcomes separately in traumatic and nontraumatic brain injury patients. To this end, we integrated etiological and outcome information using a machine learning approach. Specifically, the identification of EEG biomarkers of etiology serves to quantify the differential contribution of EEG parameters in the prediction of the clinical outcome separately in the two etiological groups. Therefore, in the two etiology groups, we investigated the accuracy of the EEG biomarkers, as identified in the first step of analyses, to discriminate DoC patients who improved vs. those who did not.

To summarize, the present study can offer several novel insights:

1. Translational value: We highlight the translational value and feasibility of EEG biomarkers based on standard clinical EEG in the assessment of functional outcome in DoC patients;
2. Methodological value: We directly compare DoC patients with different etiologies to identify those EEG biomarkers able to predict the clinical outcome in traumatic and nontraumatic DoC patients;
3. Computational value: we propose a machine learning model, based on those discriminative EEG biomarkers, for the classification of the functional outcome in DoC patients.

## 2. Materials and Methods

Step 1: To identify EEG biomarkers of different etiologies and clinical outcomes in DoC patients.

In this retrospective study, electrophysiological measures extracted from a standard clinical EEG recorded at 1 month after acute brain injury (T0) were used to discriminate the etiology of the brain injury and to predict clinical outcome 6 months after the injury (T1) in DoC patients. Specifically, we first investigated which EEG measures (i.e., qEEG and functional connectivity measures) can discriminate patients on the basis of their brain injury etiology (i.e., traumatic or nontraumatic) and the corresponding clinical outcome at 6 months (i.e., improved or nonimproved patients) to identify those EEG measures able to predict clinical changes (i.e., outcome) of the level of consciousness as measured by a specific clinical scale (i.e., the GOS) [6,43].

### 2.1. Participants

Inclusion criteria for all patients were as follows: (1) severe acquired brain injury after traumatic or nontraumatic etiologies; (2) diagnosis of disorder of consciousness (i.e., MCS or UWS) with the use of specific clinical scales (GOS < 3 or GOSE < 3 or CRS-R < 23); (3) EEG and clinical data availability at 1 and 6 months after brain injury; (4) age between 18 and 80 years old. Exclusion criteria were as follows: (1) diagnosis of locked-in syndrome (patients with LiS present total paralysis, but intact consciousness); (2) diagnosis of brain death, which implies a persistent state; (3) infectious lesions of the brain (i.e., abscess and encephalitis).

All procedures were conducted in accordance with the Declaration of Helsinki and approved by the Ethical Committee Area Vasta Emilia Centro (CE num. 841-2021-OSS-AUSLBO) Bologna, Italy. Data were acquired during routine clinical care by trained clinicians between 2005 and 2020.

### 2.2. Intervention: EEG Data Acquisition

Resting-state EEG data were recorded according to the Italian guidelines [44] for the clinical use of the EEG. A total of 19 Ag/AgCl-cup electrodes were positioned according to the 10/20 system and referenced to the linked ear lobes. Impedance for EEG and electrooculogram (EOG) electrodes were kept below 10 kΩ. EEG data were continuously recorded at a sampling rate of 1024 Hz. All electrodes were offline resampled to 500 Hz and filtered with a 1–30 Hz bandpass filter. A single EEG session lasted 20 min. Offline, EEG artefacts were eliminated using the pop_autorej function on EEGLAB [45], which automatically detects and eliminates artefact data. This function first identifies extremely large potential fluctuations in order to detect artefacts from scalp electrodes data or other unreasonably large amplitude events. Then, it rejects data epochs containing data values outside a given standard deviation (3 SD). Lastly, the EEG data were re-epoched in segments of 1 s with the function pop_repoch on EEGLAB [46], and linear trends were corrected with the function ‘detrend’ on EEGLAB.

### 2.3. Control

No control for the intervention was planned as all participants underwent EEG assessment. However, comparisons between patients with different etiologies and clinical outcomes were planned.

### 2.4. Outcome Measures

#### 2.4.1. Clinical Measures

Patients’ demographic information and the date of the brain injury were collected and reported in specific case report forms. Moreover, brain injury etiologies were used to distinguish between traumatic and nontraumatic brain injury patients.

The Glasgow Outcome Scale (GOS) [14,21,47,48,49], a specific scale used to discriminate between different levels of functional outcome in DoC patients, was administered 1 month and 6 months after the brain injury. All patients underwent usual care during the 6 months after brain injury. The difference between the GOS score at 1 month after brain injury (T0) and the score at 6 months after brain injury (T1) was used to estimate changes in the functional outcome (i.e., clinical outcome). Score differences were used to distinguish patients with an improvement in the clinical outcome (GOS score difference of at least +1 point between T0 and T1) from those with a lack of improvement in the clinical outcome (i.e., those who did not show any change in the GOS score between T0 and T1 or an impairment) [21,50]. As the GOS directly assesses the functional outcome in terms of consciousness and disability, it is a suitable clinical test to reach our goal to predict clinical outcome in DoC patients [14,48,51,52,53,54,55].

#### 2.4.2. Quantitative EEG Measures

Four quantitative EEG (qEEG) measures were extracted: z-scored power spectral density, dominant frequency peak, permutation entropy, and mean amplitude. First, power spectral data from the averaged electrodes and epochs were calculated using the “pop_spectopo” function on EEGLAB. The power spectral data expressed in μv^2^/Hz were transformed in z points to identify the frequency peak (i.e., dominant frequency) in Hertz (Hz) and the power spectral density (z-scored PSD) divided into specific frequencies: delta (1–3 Hz), theta (4–7 Hz), alpha (8–13 Hz), and beta (14–30 Hz) [38,56]. The power was calculated as the mean value in each frequency band. Furthermore, in order to evaluate permutation entropy (i.e., PeEn) on single electrodes and on the average of all electrodes, the “pec” function of EEGLAB was applied to the EEG data [33]. Similarly, the “mean” function was applied to estimate the mean amplitude (Amp) in μv [23].

#### 2.4.3. Functional Connectivity Measures

EEG-based functional connectivity estimates the association between electrode signals. There are several methods for quantifying functional connectivity on the basis of EEG data [20,21]. In particular, methods can be based on associations between phases (e.g., weighted phase lag index), between power in specific frequency bands (e.g., partial coherence), and in the complexity of the EEG signal (e.g., mutual information). For the connectivity analyses, the EEG signal was first filtered with a spatial filter (i.e., Laplacian filter) [57]. The Laplacian filter allows subtracting the activity of contiguous electrodes from each electrode and reduces the risk of false positive connectivity due to the effects of common neural sources on contiguous electrodes. The connectivity indices were calculated for each pair of electrodes, which resulted in 19 × 19 connectivity matrices. Connectivity measures were then extracted in specific regions of interest (ROI): right frontoparietal, left frontoparietal, frontal interhemispheric, central interhemispheric, and posterior interhemispheric ROIs.

In the present study, three functional connectivity measures were considered: (a) weighted phase lag index (wPLI), (b) partial coherence (PCoh), and (c) mutual information (MI).

(a) For the weighted phase lag index (wPLI), the time–frequency data were first calculated via convolution with complex Morlet wavelets. Convolution was performed via frequency-domain multiplication [58,59]. In order to prevent the artefact of the “edges”, the signal was re-epoched in epochs of 2 s. The wPLI evaluates the consistency of the phase differences between two timeseries [60] (e.g., EEG signal over specific electrodes). The wPLI was calculated on the individual EEG dominant frequency [46]. wPLI values can range from 0 to 1, where a higher value indicates a relationship between the phases of two signals. A correct threshold for each participant was set to detect residual false positive connectivity [61].
(b) The partial coherence (PCoh) values were calculated on the entire EEG signal with the “pop_newcrossf” function on EEGLAB and on the individual EEG dominant frequency (to improve frequency resolution, the pad ratio parameter was set to 8) [62]. Absolute correlations were extracted for each pair of electrodes and corrected for multiple comparisons. The PCoh values can range from 0 to 1. Larger values indicate a stronger relationship between the two signals at a specific frequency.
(c) The mutual information (MI) is a functional connectivity index that estimates the level of information shared between two variables or time series. The MI is calculated by adding the individual entropies (H) of the two timeseries and subtracting the joint entropy. For MI analyses, data were first divided into 10 bins on the basis of the Freedman–Diaconis rule [58]. The MI values were extracted with the “mutualinformationx” function on MATLAB for each pair of electrodes. Higher values indicate higher levels of information shared between two signals in terms of oscillations and similarity in the waveforms.

### 2.5. Statistical Analyses

For the first step, between-group statistical analyses were performed to identify EEG biomarkers of different etiologies and clinical outcomes. Specifically, analyses were separately performed on two between-subject factors: etiology (traumatic (TBI) vs. nontraumatic (non-TBI) etiologies) [21] and clinical outcome (improved vs. nonimproved patients) [21,22,23,63]. For both etiology and clinical outcome, we employed the same analytical strategy. In particular, mixed-model ANOVAs with repeated measures on the between-subject variable group (TBI vs. non-TBI for factor etiology and improved vs. nonimproved patients for the factor clinical outcome) were performed. For the functional connectivity indices, the additional within-subject variable ROI (right frontoparietal, left frontoparietal, frontal interhemispheric, central interhemispheric, and posterior interhemispheric) was considered. Between-group planned comparisons were performed using two-tailed *t*-tests with 1000 bootstrap corrections. To compensate for violations of sphericity in the ANOVA, Greenhouse–Geisser corrections were applied [64], and corrected *p*-values were reported. Effect sizes were estimated with partial eta squared (ηp^2^) and Cohen’s d (d) for between-group comparisons. A preliminary Shapiro–Wilk test for normality distribution was performed for all measures [65]. For similar statistical procedures, see also [66,67,68,69,70].

Step 2: Accuracy of EEG biomarkers to predict clinical outcome in traumatic and nontraumatic brain injury.

For the second step, we integrated results from step 1 using a machine learning procedure (see also [46]) to maximize the informative value provided by the combinations of EEG biomarkers in predicting the clinical outcome at the level of the individual patient for the TBI and non-TBI groups.

#### 2.5.1. Features Extraction and Data Aggregation

The initial total number of features examined in aim 1 was 43. The most discriminative features were then selected on the basis of the statistical analyses and results of step 1. In particular, for the EEG feature extraction procedure, we extracted the following information from each participant’s recording: qEEG features (dominant frequency, permutation entropy, mean amplitudem and delta, theta, alpha, and beta zPSD) and functional connectivity features (wPLI, PCoh, and MI). The selected features were aggregated in matrices where the rows represented participants (i.e., instances) and the columns represented the values of the features. The instances were used to feed a machine learning algorithm, as explained in the next section.

#### 2.5.2. Classification Method

For this step of the analysis, a stepwise linear discriminant analysis (LDA) was applied with a leave-one-subject-out cross-validation. The goal of LDA is to discriminate two classes of data in low-dimensional space by retaining the features with the higher discriminative power. LDA was already used in previous research studies, and it was recommended by the International Federation of Clinical Neurophysiology for EEG research [71] and for the assessment of DoC [72,73]. However, we additionally used a traditional multivariate logistic regression as a control for the LDA as suggested in previous studies [74,75,76]. Within the leave-one-subject-out cross-validation, each feature array was used once as validation data, with the remaining data as the training data [3,77]. Then, the percentage of correctly classified instances was calculated. While this percentage reflects the classification accuracy, we also calculated sensitivity and specificity. For the classification, we used the clinical outcome (i.e., improved vs. nonimproved patients) as a categorical grouping variable and the etiology (i.e., TBI vs. non-TBI) as a factor. In this way, the accuracy of EEG biomarkers of the clinical outcome was estimated separately for TBI and non-TBI patients. Moreover, given the retrospective nature of the study, we could deal with imbalanced classifiers [78]; thus, we calculated additional metrics, such as the balanced accuracy (the average of sensitivity and specificity) and the precision (the number of positive class predictions divided by the sum of true-positive and false-positive instances). Only EEG variables that showed sensitivity to distinguish between groups in the analyses performed to address the first step entered the LDA.

It is important to note that, for the study purpose, we needed an independent clinical indicator (i.e., the GOS) which could discriminate between two groups of patients, with a relative lower and higher outcome. In other words, we needed a binary indicator to show the potential of EEG biomarkers to discriminate individuals on the basis of their outcome, without any pretense to make an accurate diagnosis of the level of consciousness for each individual. Thus, for the purpose of the study, we regard the GOS a sufficient and adequate clinical indicator [79].

#### 2.5.3. Sample Size Estimation and Statistical Software Employed

The preliminary sample size for step 1 was calculated with the G*Power software, version 3.1 (Heinrich Heine University Düsseldorf, Düsseldorf, Germany) [80]. Parameters used in the analyses were derived from previous studies on the primary endpoint z-score PSD on the alpha band [22]. The following parameters were used: α (two-tailed) = 0.05 (threshold probability for rejecting the null hypothesis; type I error rate), β = 0.2 (probability of failing to reject the null hypothesis under the alternative hypothesis), and ηp^2^ = 0.56 (effect size calculated on preliminary studies). The minimum estimated sample size, using these parameters, was 28 subjects.

All statistical analyses for steps 1 and 2 were performed with the SPSS software (IBM Corp., Armonk, NY, USA) (Version 13). The feature extraction was performed using custom-made routines in MATLAB 2015 b (The MathWorks, Inc., Natick, MA, USA) and EEGLAB (v. 13.0.1).

## 3. Results

Step 1: To identify EEG biomarkers of different etiologies and clinical outcomes in DoC patients.

### 3.1. Clinical Results

Thirty-three DoC patients (21 males aged 19–71) were included in this study. Descriptive statistics (Table 1) showed that 15 patients suffered from traumatic brain injury, while 18 patients had a nontraumatic etiology (i.e., vascular and anoxic brain injury). In particular, within the non-TBI group, nine patients suffered from an ischemic or hemorrhagic stroke while nine patients had a post-anoxic etiology. Lesion locations were extracted for each participant according to the most recent CT or MRI scan. The visual inspection of non-TBI patients’ lesion profiles showed a maximal lesion overlap over the basal ganglia and the thalamus. Fifteen patients presented diffuse axonal damages after TBI; specifically, according to the Marshall classification of traumatic brain injury [81], eight patients were classified as “diffuse injury” (IV), and seven patients were classified as “evacuated mass lesion” (V). All patients underwent usual care and received symptomatic treatments. Whenever necessary, patients were treated with decompressive craniectomy to reduce high intracranial pressure. Demographic data show that non-TBI patients were about 15 years older than TBI patients (*t*(31) = 2.62, *p* = 013, d = 0.46). At baseline, no differences emerged between groups in the severity of brain injury as revealed by the analyses of the Glasgow Coma Scale (GCS) at admission (i.e., baseline) (*t*(31) = 0.57, *p* = 575, d = 0.11). Lastly, clinical changes were comparable between the two etiological groups as the proportion of patients improved at T1, albeit numerically larger in the TBI group, was not statistically different between TBI (50%) and non-TBI patients (29.5%; independent samples Mann–Whitney U test; *p* = 215).

### 3.2. Etiology Biomarkers

**Figure 1:**
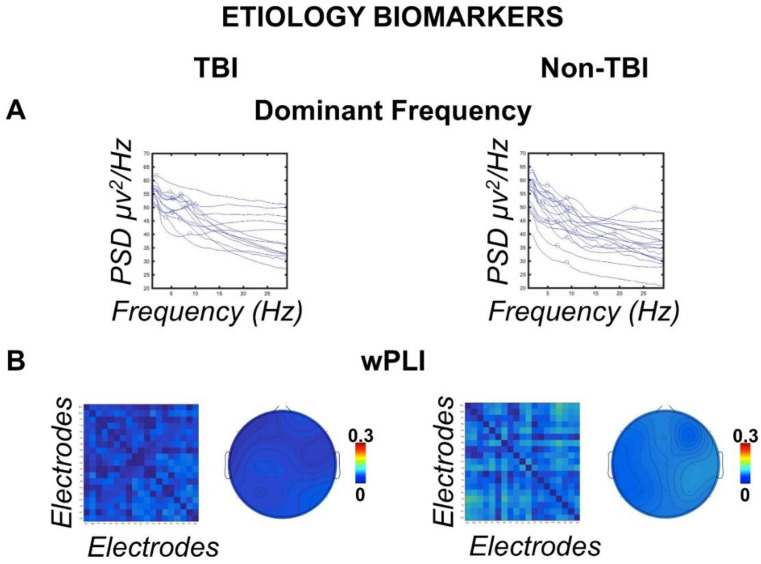
**Etiology biomarkers.** (**A**) Single-subject power spectral density (PSD) in the frequency range (1–30 Hz) divided into traumatic (TBI) and nontraumatic (non-TBI) etiologies. The circles identify the dominant frequency peaks showing a distribution of the peaks toward slower frequencies in the TBI group. (**B**) Connectivity matrices between scalp electrodes of the weighted phase lag index (wPLI) for TBI and non-TBI groups showing stronger global connectivity in the non-TBI group. Topographies show the grand mean wPLI connectomes for each electrode.

EEG data were analyzed for the between-subject factor etiology. As demographic results showed that TBI patients were younger than non-TBI patients (see above), the variable age was used as a covariate in the subsequent analyses of covariance (ANCOVAs). The ANCOVA (similar to the ANOVA) examined the effects of independent variables on dependent variables while factoring out the effect of the covariate “age”. A visual assessment of Figure 1A,B reveals the generally slower dominant frequency in TBI patients compared to non-TBI, which additionally showed stronger residual connectivity expressed by the wPLI. Statistical analyses confirmed these impressions. Specifically, qEEG results showed a faster dominant frequency (one-way ANCOVA F_(2,30)_ = 3.763, one-tailed *p* = 031, ηp^2^ = 111) in the non-TBI group (M = 5.966 Hz, standard error of the mean SE = 0.606 Hz) compared to the TBI group (M = 3.997 Hz, SE = 0.621 Hz) (*t*(31) = 2.26, *p* = 031, d = 0.39), as shown in Figure 1A. Similar analyses on the other qEEG measures did not show further significant results (all *p* ≥ 228).

Analyses on the functional connectivity indices showed larger connectivity in the wPLI for the non-TBI group (M = 053 PLI, SE = 012 PLI) in all the considered ROIs (main effect of etiology in the repeated measures ANCOVA; F_(1,30)_ = 6.862, *p* = 007, ηp^2^ = 186) compared to the TBI group (M = 047 PLI, SE = 014 PLI) (Figure 1B). No further significant between groups or interaction effects were found in the other functional connectivity indices (all F < 0.344, all *p* > 562, all ηp^2^ < 011).

### 3.3. Outcome Biomarkers

**Figure 2:**
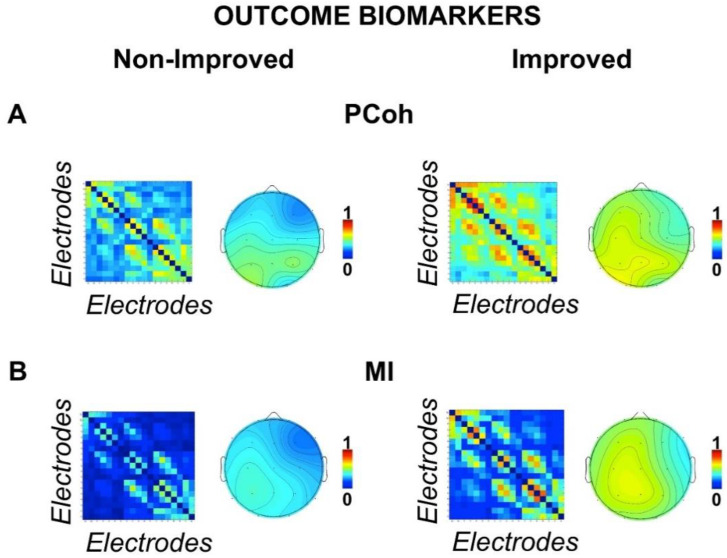
Outcome biomarkers. Connectivity matrices between scalp electrodes of the (**A**) partial coherence (PCoh) and the (**B**) mutual information (MI) for improved and nonimproved patients. Both figures show stronger connectivity for improved patients. Topographies show grand mean PCoh and MI connectomes for each electrode.

EEG data were analyzed for the between-subject factor ‘clinical outcome’ (improved vs. nonimproved patients). A visual assessment of the Figure 2A,B shows that EEG-based connectivity (expressed by the PCoh and the MI) calculated at T0 was stronger for those patients who showed at T1 functional outcome improvements. Statistical analyses confirmed these impressions. Analyses on the functional connectivity indeed showed a general stronger connectivity both for the MI (main effect of group, F_(1,31)_ = 5.36, *p* = 027, ηp^2^ = 147) and for the PCoh (main effect of group, F_(1,31)_ = 5.81, *p* = 022, ηp^2^ = 158) indices in the improved outcome group (MI mean = 326, SE = 046; PCoh mean = 464, SE = 042) compared to the nonimproved outcome group (MI mean = 188, SE = 037; PCoh mean = 333, SE = 034) Figure 2A,B. To further confirm the relative improvement in DoC patients at 6 months (T1), we ran two control analyses on the factor clinical outcome, controlling for the initial severity of the brain injury. To this aim, we first used the GCS score at baseline and then the GOS score at 1 month (T0) as covariate variables in two separate analyses of covariance (ANCOVAs). The results of the ANCOVAs with the within-subject factor ROI, the between-subject factor clinical outcome, and the covariate GCS or GOS at T0 confirmed our main findings. In particular, both analyses showed a significant effect of the between-subject factor clinical outcome for the MI (all F_(1,30)_ > 4.62, *p* < 04, ηp^2^ > 133) and PCoh (all F_(1,30)_ > 4.95, *p* < 034, ηp^2^ > 142), thus confirming again that patients with higher general connectivity at T0 have a higher probability of a better clinical outcome at T1 regardless of the initial GCS or GOS levels. No further significant between groups or interaction effects were found for the functional connectivity indices (all F < 1.138, all *p* > 339, all ηp^2^ < 035). However, main effects of the factor ROI (all F > 4.842, all *p* < 008, all ηp^2^ < 135) were found for all the functional connectivity indices (wPLI, PCoh and MI), indicating a general stronger interhemispheric connectivity (i.e., frontal, central, and posterior interhemispheric ROIs) in all groups compared to the intrahemispheric connectivity (i.e., right and left frontoparietal ROIs).

Lastly, between-group comparisons for the qEEG measures did not show any significant difference (all *p* ≥ 308).

Step 2: Accuracy of EEG biomarkers to predict clinical outcome in traumatic and nontraumatic brain injury.

### 3.4. Linear Discriminant Analysis

**Figure 3:**
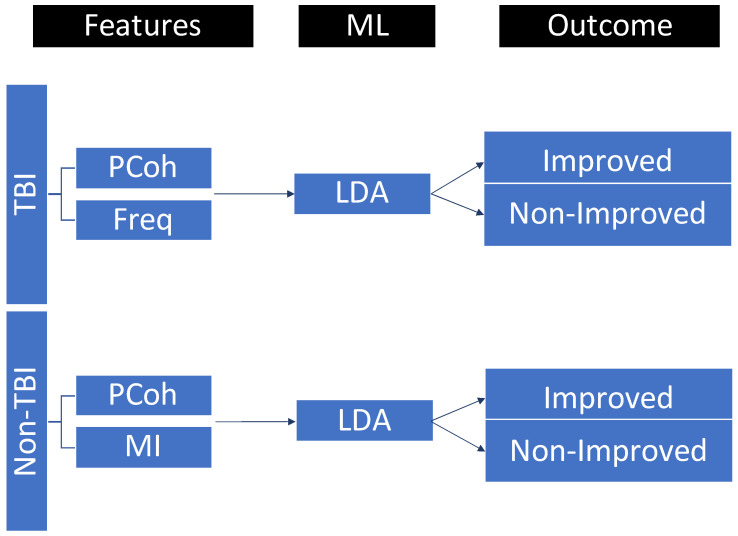
EEG features selected for traumatic (TBI) and nontraumatic (non-TBI) patients and included in the linear discriminant analysis (LDA) for clinical outcome prediction. LDA parameters were as follows: discriminant type = diagLinear (all classes had the same diagonal covariance matrix), gamma = 1, and delta = 0. PCoh = partial coherence, Freq = dominant frequency, MI = mutual information, ML = machine learning.

**Figure 4:**
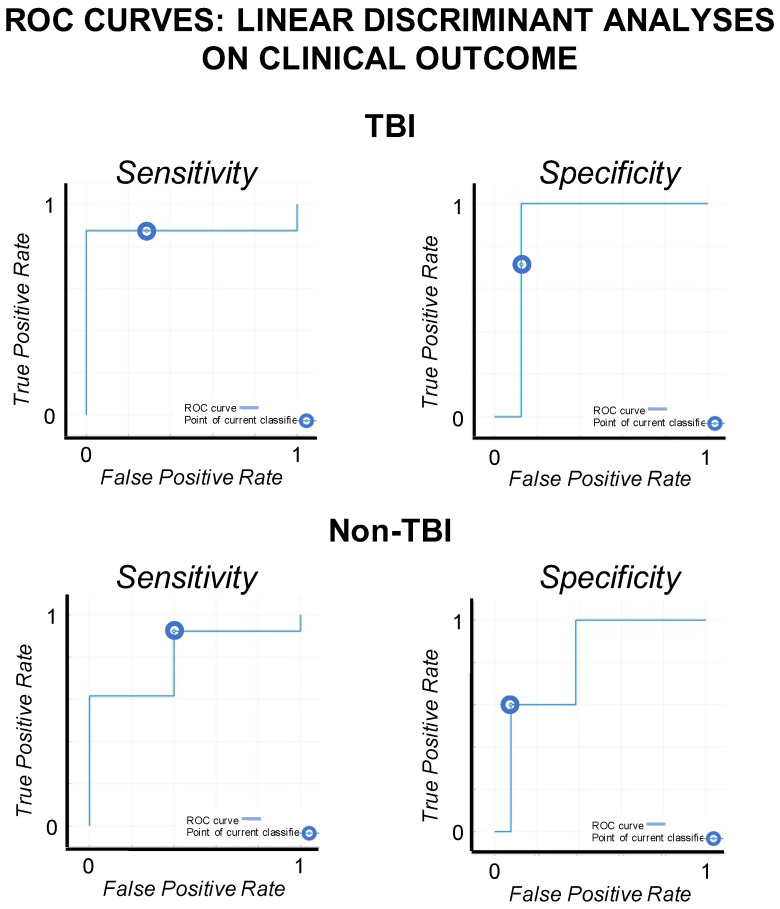
**ROC curves.** The ROC curves for sensitivity and specificity of the linear discriminant analyses on the patients’ clinical outcome (TBI vs. non-TBI patients).

Instances used for LDA analyses were 15 for TBI patients and 18 for non-TBI patients. Four features were selected for the LDA analyses on the basis of the results of step 1: one qEEG feature (i.e., dominant frequency) and three functional connectivity features (i.e., wPLI, MI, and PCoh) calculated as the mean values across ROIs. The data partition and LDA procedure are described in Figure 3. Results and ROC curves for sensitivity and specificity of the LDA are reported in Figure 4 and Table 2.

The accuracy of the EEG features to discriminate between the improved and nonimproved patients in the two etiological groups was analyzed. In the TBI group, the combination between global PCoh (i.e., mean of PCoh across ROIs) and the dominant frequency measures resulted in the best discrimination accuracy for the 6 month outcome (accuracy = 80%, balanced accuracy = 78.55%, and precision = 77.7%) with a sensitivity (i.e., patients correctly classified as nonimproved patients) of 85.7% and a specificity (i.e., patients correctly classified as improved patients) of 71.4%. The other considered measures, taken alone or in conjunction, resulted in an overall discrimination accuracy < 73.3%. In the non-TBI group, the best discrimination accuracy was evinced for the combination of two functional connectivity indices (MI and PCoh accuracy = 83.3%, balanced accuracy = 76.65%, and precision = 85.7%), calculated as the mean connectivity across ROIs, with sensitivity = 92.3% and specificity = 60.0%. The other considered measures, taken alone or in conjunction, resulted in an overall discrimination accuracy < 83.3%. Multivariate logistic regression on the same EEG features further confirmed the accuracy results of LDA in discriminating improved and nonimproved patients in both TBI (accuracy = 80%) and non-TBI patients (accuracy = 83.3%).

## 4. Discussion

The present study investigated the accuracy of psychophysiological measures extracted from standard clinical EEG for the prediction of the clinical outcome in traumatic and nontraumatic patients with DoC. Our findings described how EEG-derived measures of connectivity and qEEG measures are associated with clinical characteristics and 6 month outcomes. In particular, our results highlighted that DoC patients with a traumatic etiology show reduced dominant frequencies and lower connectivity based on wPLI compared to patients with vascular and anoxic etiologies. Furthermore, we showed that higher connectivity in the EEG brain network can predict behavioral changes in the functional outcome as measured by the GOS. Lastly, we demonstrated that the combination of different EEG features (quantitative and connectivity measures) can reliably predict 6 month clinical outcome of DoC patients after both TBI and non-TBI etiologies.

Previous studies have highlighted that quantitative EEG measures and functional connectivity within critical brain networks can provide diagnostic and predictive information for the assessment of DoC [20,21,22]. For instance, the most common EEG change associated with severe brain injury is frequency slowing with predominant delta and theta activity [45,82,83]. However, the neuropathological patterns of DoC after TBI and non-TBI are different. Several studies have reported that the pathologic substrate in DoC patients after TBI is a diffuse axonal damage, while, in non-TBI patients, lesions can be more focal especially for vascular etiologies [62,63]. In TBI, the diffuse axonal damage can cause a disconnection between cortical and subcortical structures, such as the brainstem, the thalamus, and the cerebral cortex, which are involved both in the emergence of consciousness and in the genesis of EEG rhythms [64,65], as in both cognitive (i.e., memory and learning) and social functioning [84,85]. Indeed, partial deafferentations of cortical and subcortical areas can also produce EEG rhythms within slower bands [66]. In contrast, non-TBI DoC patients with a vascular etiology show more focal cortical and subcortical lesions, with structural connectivity relatively preserved [62,63], but altered motor and behavioral abilities [86]. Thus, different neuropathological and connectivity profiles may be responsible for diverse EEG patterns. Accordingly, our results show differences in the brain patterns for TBI and non-TBI patients; thus, we can hypothesize that lower wPLI connectivity and dominant frequency in TBI presumably reflect the typical diffuse axonal damage.

Furthermore, our results confirmed that resting-state EEG-based connectivity is an accurate proxy of patients’ long-term clinical outcomes [20,21]. The notion that connectivity is important for the recovery in DoC patients after brain injury is consistent with evidence from PET [50,87,88], functional MRI [89,90,91], and high-density EEG [20,21,56]. In particular, DoC patients often show severe functional and structural thalamocortical lesions and disconnections with a drop in the EEG coherence in the damaged hemispheres [92,93]. The decrease in EEG coherence seems to be partially determined by damages of the neuronal network implicated in the emergence of consciousness [27,31,92,93] and could reflect an impairment in information sharing within the brain networks [36,94,95,96]. Indeed, levels of EEG coherence, as measured by partial coherence (PCoh) and mutual information (MI), are lower in UWS and MCS patients and in patients with a poor clinical outcome [20,36,92,97]. Accordingly, our results show that a larger levels of PCoh and of distributed information sharing (as calculated by the MI) within brain networks can support the processes needed to achieve functional improvements in DoC patients. However, while previous studies identified thalamocortical [98,99] and frontoparietal [36,100] connectivity as crucial hubs for the emergence of consciousness, we did not replicate these results here. This is probably related to the low spatial resolution of the standard clinical EEG, which provides a reduced number of scalp electrodes. Most importantly however, standard EEG protocols are easier to implement, and the accuracy of global connectivity indices to predict patients’ clinical outcome is still high (~80–83.3%) and comparable to the accuracy of other EEG settings and high-density EEG (~75–87%) [20,21,25,72]. However, the mentioned studies used different machine learning algorithms for classification and outcome prediction of DoC patients. Thus, our results cannot be directly compared with studies that used different EEG settings and statistical approaches. Importantly, here, we further validated those EEG biomarkers which can better predict functional outcome in DoC patients and excluded other biomarkers that show lower accuracy when a standard clinical EEG is used.

Lastly, it is important to notice that the etiology is an essential factor for clinicians evaluating EEG predictors of the clinical outcome in DoC patients. Specifically, while coherence in both etiological groups has a high predictive value, the global frequency of the EEG activity is a relevant feature for the prediction of the potential clinical outcome only in TBI patients. EEG features such as the dominant frequency, which presumably reflects structural damages as we and previous studies report [49], could influence the functional outcome of TBI patients [22]. Indeed, a recent study, using a standard EEG setting, showed that relative larger activity at faster frequencies, specifically in the alpha band, is a sign of potential functional improvement in TBI patients. Thus, we could hypothesize that diffuse axonal injury and the related measures are additional crucial factors to predict the outcome of DoC patients after TBI. This highlights the need for the integration of different indices and methodologies to optimize the predictive accuracy in different etiological groups.

### Study Limitations

The results of this study should be considered in light of some limitations. Different studies applied LDA for the classification of clinical outcomes in neurological, cognitive, and psychiatric conditions [101,102,103,104,105,106]. However, replication of the analysis using a larger sample size collected within prospective multicentric studies and using multiple assessment timepoints (e.g., 3 months, 6 months, and 1 year follow-up) is desirable to support the stability and generalizability of the present results. In this sense, the applicability of the standard resting-state EEG in different clinical settings represents a great advantage in terms of feasibility and replicability of the data. Furthermore, although the GOS is a frequently used coma-to-community assessment tool and is largely used for outcome prediction [20,24,43,50], it is more prone to random errors of measurement being a single-item scale. Indeed, despite the GOS being able to provide a general evaluation of the level of consciousness, it is known to be less responsive to clinical changes than summative rating scale such as the Disability Rating Scale [107]. In particular, measurement errors can be larger in TBI patients compared to non-TBI [108,109,110]. Thus, the limitation of the GOS measure could explain the lower overall accuracy of the EEG features in the prediction of the clinical outcome in TBI patients as evinced by our results. Future studies could eventually highlight the diagnostic accuracy of standard EEG using different clinical scales, such as the Coma Recovery Scale—Revised [7], as the reference for the clinical outcome.

## 5. Conclusions

This study presented three main outcomes. The most important outcome of this study allowed us to describe the advantages and limitations of a standard EEG as a tool for clinical assessment and classification of patients with DoC after severe acquired brain injury. Secondly, measures based on standard EEG, such as qEEG and functional connectivity, are promising tools for predicting and classifying DoC patients in terms of rehabilitative potentials and functional recovery. Although spontaneous functional improvements can be observed in TBI and non-TBI patients, the identification of those EEG biomarkers able to predict possible improvements is a valuable effort. Lastly, our results demonstrated that different measures extracted from the standard EEG could be beneficially combined to discriminate the patients’ potential clinical outcome using a machine learning approach. Hence, EEG biomarkers may prove highly relevant in supporting clinical and rehabilitative decision making.

## Figures and Tables

**Table 1:** Demographic and clinical data.

	TBI	Non-TBI
***N***	15	18
**Gender** Males Females	11 4	10 8
**Mean age in years (SD)**	34.3 (4.4)	49.1 (3.59)
**Glasgow Coma Scale at baseline**	4.73 (0.5)	4.22 (0.7)
**Clinical outcome at T1** Improved Nonimproved	8 7	5 13

Patients’ etiologies were divided into traumatic (TBI) and nontraumatic brain injury. Diagnostic classifications at 1 month after brain injury (T0) are reported as unresponsive wakefulness syndrome (UWS) and minimal conscious states (MCS). Clinical outcomes at 6 months after the injury (T1) are reported as improved (i.e., patients showing positive changes in the GOS > +1) or nonimproved patients (patients showing no changes or negative changes in the GOS < 0). Standard errors of the mean (SD) are in brackets.

**Table 2:** LDA results.

Etiology	Features	LDA			Clinical Outcome [95% CI]	
		Acc	Sens	Spec	Non-Improved	Improved
**TBI**	Pcoh and Freq	80.0%	85.7%	71.4%	[−1.262, 0.306]	[−0.523, 1.420]
**Non-TBI**	PCoh and MI	83.3%	92.3%	60.0%	[−1.179, 0.304]	[−0.099, 2.199]

The best accuracy results in the discrimination of the clinical outcome between improved and nonimproved patients are reported separately for traumatic (TBI) and nontraumatic (non-TBI) etiologies. The 95% confidence intervals (CI) are reported for the feature combinations. LDA = linear discriminative analyses, Acc = accuracy, Sens = sensitivity, Spec = specificity, PCoh = partial coherence, Freq = dominant frequency, MI = mutual information.

## Data Availability

The datasets used and analyzed during the current study are available from the corresponding author on reasonable request. The anonymised EEG raw data are publicly available for download at https://doi.org/10.5281/zenodo.6951440.

## Abbreviations

DoC	Disorders of consciousness
EEG	Electroencephalography
TBI	Traumatic brain injury
wPLI	Weighted phase lag index
PCoh	Partial coherence
MI	Mutual information
LDA	Linear discriminant analyses
